# Association between Systemic Immunity-Inflammation Index and Hyperlipidemia: A Population-Based Study from the NHANES (2015–2020)

**DOI:** 10.3390/nu15051177

**Published:** 2023-02-26

**Authors:** Nayili Mahemuti, Xiyue Jing, Naijian Zhang, Chuanlang Liu, Changping Li, Zhuang Cui, Yuanyuan Liu, Jiageng Chen

**Affiliations:** 1 School of Public Health, Tianjin Medical University, Tianjin 300070, China; 2 Tianjin Key Laboratory of Cerebral Vascular and Neurodegenerative Diseases, Tianjin Neurosurgical Institute, Tianjin Huanhu Hospital, Tianjin 300070, China; 3 Tianjin Key Laboratory of Environment, Nutrition and Public Health, Tianjin 300070, China

**Keywords:** systemic immunity-inflammation index, hyperlipidemia, cross-sectional study, NHANES

## Abstract

The systemic immunity-inflammation index (SII) is a novel inflammatory marker, and aberrant blood lipid levels are linked to inflammation. This study aimed to look at the probable link between SII and hyperlipidemia. The current cross-sectional investigation was carried out among people with complete SII and hyperlipidemia data from the 2015–2020 National Health and Nutrition Examination Survey (NHANES). SII was computed by dividing the platelet count × the neutrophil count/the lymphocyte count. The National Cholesterol Education Program standards were used to define hyperlipidemia. The nonlinear association between SII and hyperlipidemia was described using fitted smoothing curves and threshold effect analyses. A total of 6117 US adults were included in our study. A substantial positive correlation between SII and hyperlipidemia was found [1.03 (1.01, 1.05)] in a multivariate linear regression analysis. Age, sex, body mass index, smoking status, hypertension, and diabetes were not significantly correlated with this positive connection, according to subgroup analysis and interaction testing (*p* for interaction > 0.05). Additionally, we discovered a non-linear association between SII and hyperlipidemia with an inflection point of 479.15 using a two-segment linear regression model. Our findings suggest a significant association between SII levels and hyperlipidemia. More large-scale prospective studies are needed to investigate the role of SII in hyperlipidemia.

## 1. Introduction

Hyperlipidemia is a systemic metabolic illness defined by unusually high amounts of lipids in the blood, including cholesterol and triglycerides. Hyperlipidemia has been connected to a variety of health issues, including the combination of diabetes, obesity, and hypertension, known as metabolic syndrome, which poses major hazards to human health [1]. The consequences of hyperlipidemia on the vascular system are well established [2]. In populations in the United States, Europe, and emerging nations, hyperlipidemia is a key modifiable risk factor for developing atherosclerotic cardiovascular disease [3]. In the United States, 28 million people had total cholesterol levels higher than 240 mg/dL [4]. In addition, hyperlipidemia significantly increases the risk of cardiovascular and immune diseases and is a significant cause of stroke and death [5].

Systemic inflammation can be quantified using a variety of biochemical or hematological indicators that are regularly determined in normal blood tests or as ratios generated from these measures [6]. The systemic immunity-inflammation index (SII) is a stable new inflammatory biomarker computed from platelet count × neutrophil count/lymphocyte count [7,8]. SII could assess local systemic inflammation and the immunological response across the body [9,10]. SII is now employed as a prognostic factor in cancer investigations. The interaction between systemic inflammation and the local immune response has been identified as the seventh cancer hallmark, and it has been shown to be involved in the initiation, development, and progression of various forms of cancer [11,12]. Cervical cancer [13], esophageal cancer [14], and hepatocellular carcinoma [15] are examples. In addition to tumors, Ya et al. reported that SII has predictive value for coronary artery disease (CAD) [16].

Some studies have clarified that inflammation is associated with blood lipid levels. Ma et al. reported that higher plasma *C*-reaction protein (CRP) levels and higher urinary copper levels were associated with higher serum total cholesterol (TC), triglyceride (TG), low-density lipoprotein cholesterol (LDL-C), and lower high density lipoprotein cholesterol (HDL-C) concentrations. According to mediation analysis, CRP played a 6.27% role in the association between urinary copper and TG. These findings imply that systemic inflammation plays a role in the association between copper exposure and abnormal lipids, which may contribute to the development of dyslipidemia [17]. Natalia et al. reported that postprandial hyperlipidemia (PPHL) is more common in rheumatoid arthritis (RA) patients than in healthy controls. In individuals with rheumatoid arthritis (RA), postprandial hyperlipidemia (PPHL) is linked to inflammation and subclinical atherosclerosis [18]. Melody et al. reported that blood lipid levels appear to have a pleiotropic connection with *C*-reactive protein (CRP) [19]. In addition, Kenneth et al. reported that inflammation could alter a variety of lipid metabolisms [20]. However, the association between the inflammatory level biomarker Systemic Immunity-Inflammation Index (SII) and hyperlipidemia is not well characterized.

Therefore, we conducted a population-based cross-sectional study to investigate the relationship between systemic immunity-inflammation indices (SII) and hyperlipidemia in National Health and Nutrition Examination Survey (NHANES) adult participants.

## 2. Materials and Methods

### 2.1. Study Population

The NHANES is an ongoing survey of the US national population that employs a complex, multistage, and probabilistic sampling technique to provide a plethora of information on the nutrition and health of the US population. More information is available at http://www.cdc.gov/nchs/nhanes/index.htm (accessed on 8 February 2023) detailing the NHANES survey’s continuous design. All study procedures were authorized by the National Center for Health Statistics’ ethical review board prior to data collection, and all participants gave their signed, informed consent.

**Figure 1:**
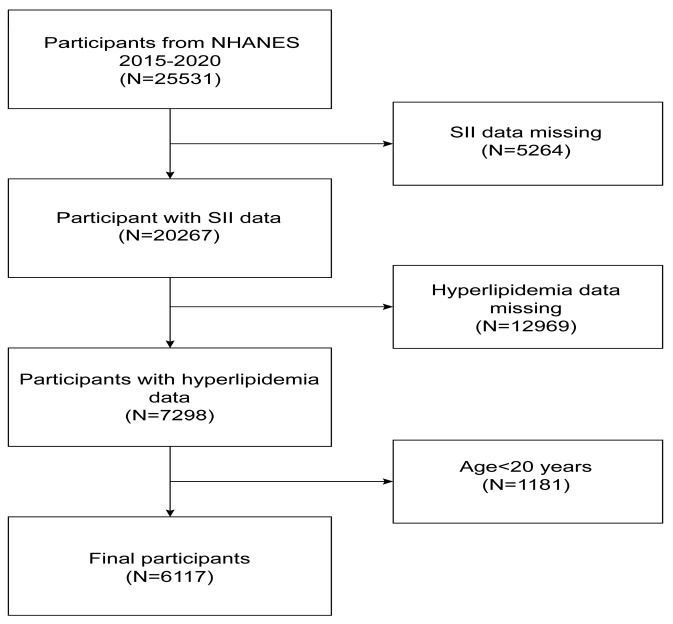
Flowchart of participant selection. NHANES, National Health and Nutrition Examination Survey.

In the investigation, we removed from the 25,531 eligible people 5264 participants with missing SII data, 12,969 participants with missing hyperlipidemia data, and 1181 participants younger than 20 years of age. The research included a total of 6117 individuals. Figure 1 depicts the sample selection.

### 2.2. Assessment of Hyperlipidemia

Adult Treatment Panel III (ATP 3) of the National Cholesterol Education Program (NCEP) classified hyperlipidemia as total cholesterol 200 mg/dL, triglycerides 150 mg/dL, HDL 40 mg/dL in males and 50 mg/dL in females, or low-density lipoprotein 130 mg/dL [21]. Alternately, persons who reported using cholesterol-lowering drugs were also classified as having hyperlipidemia.

### 2.3. SII and Covariates

The systemic immunity-inflammation index is the dependent variable in this investigation. SII was intended as an exposure variable in our research. Using automated hematology analysis equipment (a CoulterDxH 800 analyzer), the lymphocyte, neutrophil, and platelet counts were measured and reported as 10^3^ cells/mL. The SII level was determined by multiplying the platelet count by the neutrophil count/lymphocyte count [7,22,23]. Based on prior studies, possible confounding factors linked with SII and hyperlipidemia were included in the final analysis [24]. Covariates included age, race, sex, education level, income-to-poverty ratio, marital status, drinking status, smoking status, BMI, hypertension, and diabetes. Among them, race was categorized as Mexican American, Non-Hispanic White, Non-Hispanic Black, other Hispanic, other race. The levels of education were designated as less than high school, high school, and more than high school. On a scale from 1.5 to >3.5, the income to poverty ratio was divided into three categories: 1.5, 1.5–3.5, and >3.5 [25]. Three categories of marital status were identified: married/living with a partner, widowed/divorced/separated, never married. Drinking status was categorized as excessive alcohol consumption, moderate alcohol consumption, or light alcohol consumption. Three drinks per day for women and four drinks per day for men were considered as excessive alcohol consumption. The definition of moderate alcohol consumption was two drinks per day for women and three drinks per day for men. Other alcohol consumption was deemed light [26]. Smoking status was categorized as either now smoking, formerly smoking, or never. Never smokers were defined as having smoked no more than 100 cigarettes in their lives, ex-smokers as having smoked more than 100 cigarettes but no longer smoking, and current smokers as having smoked more than 100 cigarettes but sometimes or consistently. <25 kg/m^2^, 25 to 30 kg/m^2^, >30 kg/m^2^ BMI categories were established [27]. Average blood pressure >140 mmHg systolic and/or 90 mmHg diastolic, as reported by a physician diagnosed with hypertension or using hypertensive medication, was used to characterize hypertension. Diabetes was defined as the reporting of a diabetic diagnosis and the use of diabetes medicine or insulin.

### 2.4. Statistical Analysis

SII was divided into quartiles from lowest (Q1) to highest (Q4); continuous variables were expressed as means with standard deviations (SDs) and categorical variables as proportions; the differences between participants grouped by SII quartiles and the differences between participants with or without hyperlipidemia were assessed using a weighted *t*-test (continuous variables) or a weighted chi-square test (categorical variables). To examine the association between SII and hyperlipidemia, multivariate logistic regression analysis between SII and hyperlipidemia was used to construct multivariate tests, using three models with no covariates in model 1; model 2 was adjusted for age, sex, and race; model 3 was adjusted for age, sex, race, marital status, income to poverty ratio, education level, drinking status, smoking status, BMI, hypertension, and diabetes; and SII and hyperlipidemia were evaluated using odds ratios (OR) and 95% confidence interval (CI) in the models. Using three models, multivariate tests were constructed by controlling for variables and fitting a smooth curve. Using a threshold effects analysis model, the association and inflection points between SII and hyperlipidemia were investigated. Finally, the same statistical analysis procedures outlined before were used for the subgroup based on sex. The statistical analyses were conducted using R studio (Version 4.2.2) and EmpowerStats (version 2.0). A *p*-value < 0.05 was determined to be significant. We used a weighting strategy to lessen the substantial volatility of our dataset.

## 3. Results

### 3.1. Baseline Characteristics of Participants

There were 6117 participants enrolled, of whom 48.08% were male, with an average age of 50.70 ± 17.43 years. The mean SII ± SD concentrations were 459.54 ± 317.28. There were 69.72% of participants have hyperlipidemia. 

The clinical characteristics of the participants according to hyperlipidemia as a column-stratified variable are shown in Table 1. The presence or absence of hyperlipidemia was statistically significant with age, sex, race, education level, marital status, BMI, drinking status, smoking status, hypertension, diabetes, and SII (*p* < 0.05). Compared with non-hyperlipidemia, patients with hyperlipidemia tended to be older, female, non-Hispanic white, possess more high school education, married/living with partner, 0 < BMI < 25 kg/m^2^, light alcohol consumers, never smokers, and without diabetes or hypertension, as well as having higher levels of SII.

The clinical characteristics of the participants according to the quartiles of SII are shown in Table 2. There was statistically significant difference among the SII quartiles in terms of age, sex, race, marital status, BMI, drinking status, smoking status, hypertension, diabetes, and hyperlipidemia (*p* < 0.05). Participants who fell into the Quartile 4 group tended to be older, female, non-Hispanic white, married/living with a partner, a BMI > 30 kg/m^2^, light alcohol consumption, never smokers, with no diabetes, hypertension, and with hyperlipidemia.

### 3.2. Association between SII and Hyperlipidemia

Because the effect value is not apparent, SII/100 is used to amplify the effect value by 100 times. Table 3 showed the results of the multivariable regression analysis between SII/100 and hyperlipidemia. This association was significant both in model 1 (1.04 (1.02, 1.06)) and model 2 (1.03 (1.01, 1.05)). However, in model 3, the positive association between SII and hyperlipidemia became insignificant (1.02 (1.00, 1.04)). Sensitivity analysis was performed with SII quartiles, and the ORs for Q1, Q2, Q3, and Q4 in model 2 were 1.00, 1.05 (0.90, 1.23), 1.31 (1.12, 1.54), and 1.27 (1.08, 1.50), respectively, compared to Quartile 1, participants in Quartile 4 had an association with 27.04% increased risk of hyperlipidemia (*p* for trend < 0.05).

**Figure 2:**
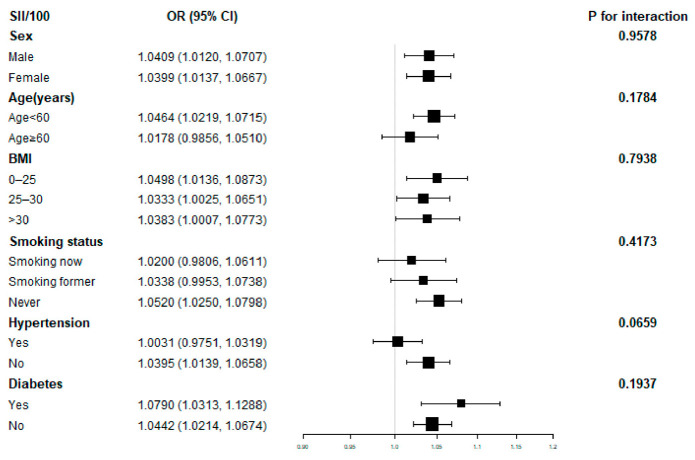
Subgroup analysis for the association between SII and hyperlipidemia.

Further subgroup analysis revealed that the association of SII with hyperlipidemia was not consistent, as shown in Figure 2. SII was shown to correlate significantly with hyperlipidemia in subgroups stratified by sex, BMI, and diabetes (*p* < 0.05). Interaction tests revealed that the relationship between SII and hyperlipidemia was not statistically different across strata, showing that age, sex, BMI, smoking status, hypertension, and diabetes did not significantly impact this positive correlation (*p* for interaction> 0.05). 

**Figure 3:**
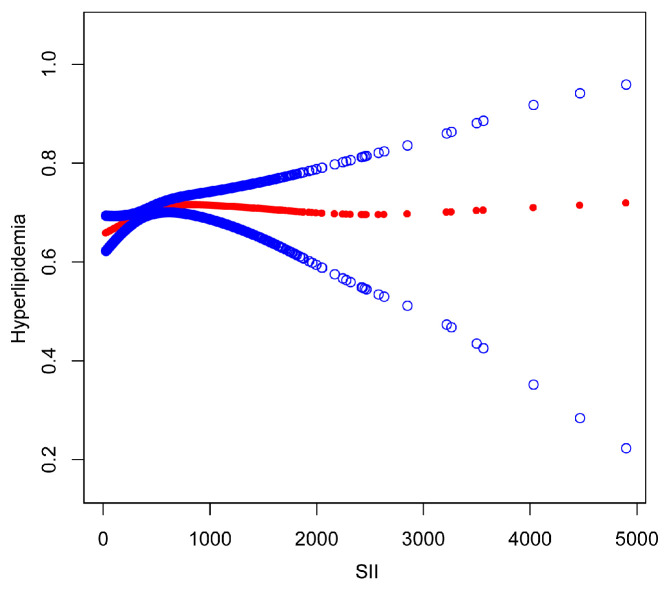
The association between SII and hyperlipidemia. The solid red line represents the smooth curve fit between variables. Blue bands represent the 95% confidence interval from the fit.

**Figure 4:**
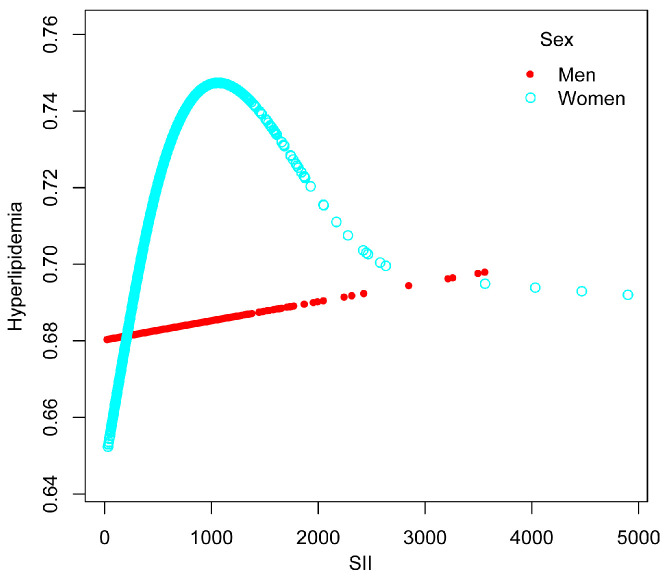
The association between SII and hyperlipidemia stratified by sex.

The nonlinear association between SII and hyperlipidemia was then described using smoothed curve fitting (Figure 3 and Figure 4). Adjusted variables: age, sex, race, education level, marital status, BMI, drinking status, smoking status, hypertension, and diabetes. We discovered a nonlinear relationship between SII and hyperlipidemia using a two-stage linear regression model with an inflection point of 479.15. Adjusted variables: age, race, education level, marital status, BMI, drinking status, smoking status, hypertension, and diabetes. In women, an inverted U-shaped curve with an inflection point of 958.14 was detected after stratified analysis by sex, as shown in Table 4.

## 4. Discussion

In our cross-sectional study, we discovered that higher SII was associated with a higher risk of hyperlipidemia. The results of the subgroup analyses and interaction testing indicated that this connection was similar across populations. An inverted U-shape relationship between SII and hyperlipidemia was also discovered, with an inflection point of 479.15. The data mentioned above imply that when SII is below 479.15, SII is an independent risk factor for hyperlipidemia.

To our knowledge, this is the first investigation on the relationship between SII and hyperlipidemia. The relationship between SII levels and blood lipids has been observed in previous epidemiological studies. For example, According to Zhu et al., the observed connection between ethylene oxide(EO) exposure and serum lipid profiles is mediated by systemic inflammation. Inflammatory indicators substantially mediated the links between hemoglobin adducts of HbEO and HDL-C and TG at the highest mediated proportions of 21.40% and 33.40%, respectively [28]. A study from rural northeast China shows that subjects with high LDL-C levels had higher levels of inflammatory markers overall. SII was also considerably higher in patients with low HDL-C [29]. Wei et al. discovered that lipid profiles were linked with neutrophils, lymphocytes, monocytes, and platelets, revealing a possible association between SII and abnormal blood lipid levels [30]. A cross-sectional study of 2631 participants in the East Coast city of Fujian Province showed that in male adults, five types of dyslipidemia increased circulation levels of IL-6, TNF-, and MCP-1 in male adults compared to the standard lipid group, and that dyslipidemia was associated with an altered inflammatory state [31]. According to several research studies, patients with inflammatory disorders have been shown to have aberrant blood lipid levels. Moreover, it has been discovered that individuals with Sjögren’s syndrome, inflammatory bowel disease, and ankylosing spondylitis have decreased HDL-C levels [32,33,34,35]. LDL-cholesterol (LDL-C) and triglyceride levels varied, although LDL-C levels tended to be lower and triglyceride levels tended to be higher. A case-control study from China discovered that very low LDL-cholesterol (VLDL-C), triglycerides (TG), the VLDL/LDL cholesterol ratio, the total/HDL cholesterol ratio, and the LDL/HDL cholesterol ratio were higher in polymyositis (PM) patients than in healthy individuals, indicating that dyslipidemia is a common feature in PM patients, characterized by high-density lipoprotein cholesterol (HDL-C) and elevated triglycerides (TG). The inflammatory condition of PM may be responsible for HDL-C metabolism [36]. Our study identified a positive linear correlation between SII levels and hyperlipidemia in models 1 and 2. An inverted U-shape association between SII levels and hyperlipidemia was also discovered, with a breakpoint of 479.15. There was a positive link on the left side of the breakpoint measurement. Still, no relationship was identified on the right side, indicating a substantial threshold impact of SII and hyperlipidemia. In summary, there have been several reports of an association between inflammation and blood lipid levels. Our findings confirm prior research suggesting high SII levels have an association with increasing the risk of hyperlipidemia.

The probable mechanisms behind this positive relationship between inflammation and abnormal blood lipid levels are not well elucidated. Wen et al. reported that STING signaling is important in mediating lipotoxicity-induced endothelial inflammation and injury, that IRE1-XBP1 signaling enhances STING signaling, that hyperlipidemia induces a pro-inflammatory response in retinal endothelial cells by activating expression of the STING pathway and signaling activation of IRE1-XBP1, and that other studies have confirmed STING’s pro-inflammatory function. Liu et al. described infantile-onset STING-associated vasculopathy caused by a systemic gain-of-function mutation in the TMEM173 gene and in patients characterized by systemic inflammation [37,38]. In addition, it has been reported that high-density lipoproteins play an important role in inflammation. Methionine sulfoxidation of apoA-I leads HDL to become pro-inflammatory via inducing pro-inflammatory cytokine production (TNF and IL-6) in mouse bone marrow-derived macrophages and mouse monocytes [39,40]. Many laboratory studies in a variety of human illness situations support the notion that statin therapy stimulates the synthesis of resolvins (SPMs), which can reduce and resolve inflammation [41,42]. SPMs work on PMNs and macrophages separately to drive resolution, making them multitarget agonists. Resolvins and all SPMs have stereochemically selective actions, which are supported by their capacity to activate receptors (G-protein-coupled receptors (GPCR)) that enhance and transmit their tissue response. This is a reflection of their production processes [43]. The ability of SPM to limit leukocyte infiltration and counter-regulate the production of pro-inflammatory mediators is one of its main biological functions [44]. The biological function of SPM in many chronic inflammatory diseases has also been demonstrated such as, periodontitis, a prevalent persistent inflammatory disorder that causes extensive periodontal damage. SPMs have been shown in several periodontal disease studies to play a significant role in controlling periodontal inflammation by restricting leukocyte trafficking to periodontal locations and decreasing the generation of pro-inflammatory mediators [45,46]. In recent studies, it has been discovered that the levels of lipoxin A4(LXA4), protectin D1(PD1), and maresin 1(MaR1) in the salivary tissues of people with periodontal inflammation are related to the progression of the illness. Interestingly, PD1 and MaR1 levels were shown to be positively connected, whereas LXA4 levels were found to be negatively associated with illness severity. Their results suggest that SPM biosynthesis pathways, or possibly their degradation routes, are controlled differently during illness, most likely as a host immune response to counteract ongoing inflammatory processes [47]. SPM biological processes have also been linked to allergy-related diseases such as allergic rhinitis and asthma. Recent studies have demonstrated that resolvin E3(RvE3) reduces the total amount of inflammatory cells and eosinophils recruited into the lungs of mice sensitized to and challenged with household dust mites. Moreover, this mediator reduced the amounts of IL-23 and IL-17 in lavage fluid and suppressed the expression of IL-23 and IL-17A mRNA in the lung and peribronchial lymph nodes. In a mouse model of allergic asthma, RvE1 decreased leukocyte recruitment into the lung and downregulated the production of pro-inflammatory cytokines in lavage fluids and macrophages [48]. Similar effects are described for metformin, which is used by diabetic patients. Metformin, a biguanide, is the most widely used diabetes medication. Metformin not only lowers chronic inflammation by improving metabolic parameters, but it also has direct anti-inflammatory action, according to recent research. A physiological dosage of metformin (100 M) was shown to inhibit Th17 inflammation in CD4 T cells from older individuals via an autophagy-dependent mechanism [49]. T cells from older individuals showed higher oxygen consumption rates (OCR), although metformin induced autophagy and reduced ROS in these cells. Previous studies in T cells from younger persons showed that autophagy suppression, driven by siRNA targeting the autophagy protein Atg3, recapitulated the respiratory and inflammatory characteristics of T cells from older individuals. Younger participants’ autophagy-deficient T cells produced no inflammatory cytokines, supporting the hypothesis that metformin lowers age-related inflammation by promoting autophagy [50].

Our investigation has several advantages. Firstly, our study’s reliability and representativeness were enhanced by a large sample size and suitable covariate correction. Sensitivity analysis reduces the possibility of false positives. However, this investigation also has limitations. Cross-sectional study designs do not allow us to identify causation, and high sample numbers of prospective studies are required to elucidate causality. Although we controlled for certain confounders, other confounding factors, such as a history of long-term use of medicines such as steroids, may still have an impact on the outcomes. Because these factors were not recorded in the NHANES, we were unable to use them in our analysis. However, the interaction between inflammation and illness is complex. Therefore, generalizing our findings may be improper.

## 5. Conclusions

Our findings suggest a significant association between SII levels and hyperlipidemia. However, the results could not establish a causal relationship, and further extensive prospective studies are needed.

## Figures and Tables

**Table 1:** Weighted characteristics of the study population based on hyperlipidemia.

	Hyperlipidemia	Non-Hyperlipidemia	*p*-Value
	N = 4265	N = 1852	
Age(years)	52.04 ± 16.37	41.30 ± 16.40	<0.001
Sex (%)			0.034
Men	47.14	51.17	
Women	52.86	48.83	
Race/Ethnicity (%)			<0.001
Mexican American	8.51	8.64	
Non-Hispanic White	65.46	60.41	
Non-Hispanic Black	9.25	14.06	
Other Hispanic	6.92	6.92	
Other Race	9.86	9.97	
Education (%)			0.007
Less than high school	5.37	4.20	
High school	8.42	6.69	
More than high school	86.20	89.10	
Marital status (%)			<0.001
Married/Living with partner	66.02	57.72	
Widowed/Divorced/ Separated	20.29	14.43	
Never married	13.68	27.85	
Income to poverty ratio (%)			0.231
0–1.5	20.42	21.97	
1.5–3.5	39.13	37.13	
>3.5	40.45	40.90	
BMI (kg/m^2^) (%)			<0.001
0–25	16.54	34.85	
25–30	47.37	42.34	
>30	36.09	22.80	
Drinking status (%)			<0.001
Excessive alcohol consumption	15.34	19.56	
Moderate alcohol consumption	16.34	20.39	
Light alcohol consumption	68.32	60.05	
Smoking status (%)			<0.001
Smoking now	18.02	16.22	
Smoking former	29.10	22.60	
Never	52.87	61.18	
Hypertension (%)			<0.001
Yes	40.61	21.10	
No	59.39	78.90	
Diabetes (%)			<0.001
Yes	24.15	14.66	
No	75.85	85.34	
SII	467.67 ± 332.58	416.94 ± 264.87	<0.001
			

Mean ± SD for continuous variables: the *p*-value was calculated by weighted linear regression model. % for categorical variables: the *p*-value was calculated by a weighted chi-square test. BMI, body mass index; SII, systemic immune-inflammation index.

**Table 2:** Weighted characteristics of the study population based on SII quartiles.

	SII Quartiles				*p*-Value
	Q1	Q2	Q3	Q4	
	N = 1529	N = 1529	N = 1529	N = 1530	
Age (years)	48.99 ± 16.91	46.02 ± 17.13	49.18 ± 16.93	50.40 ± 17.21	<0.001
Sex (%)					<0.001
Men	52.77	51.36	48.88	40.49	
Women	47.23	48.64	51.12	59.51	
Race/Ethnicity (%)					<0.001
Mexican American	7.10	10.51	9.23	7.54	
Non-Hispanic White	60.07	61.15	64.86	69.52	
Non-Hispanic Black	15.09	11.31	8.65	7.75	
Other Hispanic	6.71	6.88	6.92	7.17	
Other Race	11.03	10.15	10.33	8.02	
Education (%)					0.392
Less than high school	5.60	5.30	4.32	4.78	
High school	8.00	7.88	7.00	8.66	
More than high school	86.40	86.82	88.68	86.56	
Marital status (%)					0.011
Married/Living with partner	63.91	62.63	64.15	62.97	
Widowed/Divorced/Separated	17.03	17.05	19.90	19.88	
Never married	19.07	20.33	15.95	17.16	
Income to poverty ratio (%)					0.095
0–1.5	20.55	21.71	19.25	22.18	
1.5–3.5	37.36	39.85	37.93	39.00	
>3.5	42.09	38.44	42.82	38.82	
BMI (kg/m^2^) (%)					<0.001
0–25	15.08	27.14	23.79	23.67	
25–30	73.38	42.58	35.95	29.67	
>30	11.55	30.29	40.25	46.65	
Drinking status (%)					<0.001
Excessive alcohol consumption	12.81	17.46	17.91	18.69	
Moderate alcohol consumption	17.22	18.63	17.05	17.61	
Light alcohol consumption	55.62	58.51	55.16	52.74	
Smoking status (%)					0.002
Smoking now	17.03	14.79	17.12	20.78	
Smoking former	27.35	26.70	27.73	26.48	
Never	55.62	58.51	55.16	52.74	
Hypertension (%)					<0.001
Yes	25.09	32.06	39.02	42.25	
No	74.91	67.94	60.98	57.75	
Diabetes (%)					<0.001
Yes	37.39	15.90	14.98	15.35	
No	62.61	84.10	85.02	84.65	
Hyperlipidemia (%)					<0.001
Yes	63.99	65.01	73.68	72.37	
No	36.01	34.99	26.32	27.63	
					

Mean ± SD for continuous variables: the *p*-value was calculated by a weighted linear regression model. % for categorical variables: the *p*-value was calculated by a weighted chi-square test. Q, quartile; BMI, body mass index.

**Table 3:** The association between SII and hyperlipidemia.

	Crude Model (Model 1)	Partially Adjusted Model (Model 2)	Fully Adjusted Model (Model 3)
	OR (95% CI) *p*-Value	OR (95% CI) *p*-Value	OR (95% CI) *p*-Value
SII/100	1.04 (1.02, 1.06) ***	1.03 (1.01, 1.05) *	1.02 (1.00, 1.04)
SII quartiles			
Quartile 1	Reference	Reference	Reference
Quartile 2	1.03 (0.88, 1.19)	1.05 (0.90, 1.23)	1.09 (0.92, 1.29)
Quartile 3	1.36 (1.17, 1.59) ***	1.31 (1.12, 1.54) **	1.31 (1.10, 1.55) **
Quartile 4	1.40 (1.20, 1.63) ***	1.27 (1.08, 1.50) **	1.18 (0.99, 1.41)
*p* for trend	<0.0001	0.0009	0.0416

Model 1, no covariates were adjusted. Model 2, age, sex, and race were adjusted. Model 3, age, sex, race, marital status, income to poverty ratio, education level, drinking status, smoking status, BMI, hypertension, and diabetes were adjusted. 95% CI, 95% confidence interval; OR, odds ratio; SII, systemic immunity-inflammation index. * *p* < 0.05, ** *p* < 0.01, *** *p* < 0.001; a *p* < 0.05 was considered statistically significant.

**Table 4:** Threshold effect analysis of SII on hyperlipidemia using a linear regression model.

	Adjusted OR (95% CI), *p* Value
SII	
Inflection point	479.15
SII < 479.15	1.0008 (1.0003, 1.0013) **
SII ≥ 479.15	0.9999 (0.9996, 1.0002)
Log likelihood ratio	0.008
Men	
Inflection point	112.35
SII < 112.35	1.0056 (0.9965, 1.0147)
SII ≥112.35	1.0001 (0.9997, 1.0004)
Log likelihood ratio	0.243
Women	
Inflection point	958.14
SII < 958.14	1.0006 (1.0002, 1.1010) **
SII ≥ 958.14	0.9997 (0.9992, 1.0002)
Log likelihood ratio	0.013

Age, sex, race, marital status, income-to-poverty ratio, education level, drinking status, smoking status, BMI, hypertension, and diabetes were adjusted. 95% CI, 95% Confidence Interval; OR, Odds Ratio; SII, systemic immune-inflammation index. ** *p* < 0.01, a *p* < 0.05 was considered statistically significant.

## Data Availability

All NHANES data for this study are publicly available and can be found here: https://wwwn.cdc.gov/nchs/nhanes (accessed on 9 February 2023).
